# Sequential Analysis of Oxidative Stress Markers and Vitamin C Status in Acute Bacterial Osteomyelitis

**DOI:** 10.1155/2014/975061

**Published:** 2014-08-10

**Authors:** Rade Grbic, Dijana J Miric, Bojana Kisic, Ljiljana Popovic, Vojkan Nestorovic, Aleksandar Vasic

**Affiliations:** ^1^Clinics for General and Orthopedic Surgery, Medical Faculty Pristina, Anri Dinana bb, 38220 Kosovska Mitrovica, Serbia; ^2^Institute of Biochemistry, Medical Faculty Pristina, Anri Dinana bb, 38220 Kosovska Mitrovica, Serbia; ^3^Institute of Pathophysiology, Medical Faculty Pristina, Anri Dinana bb, 38220 Kosovska Mitrovica, Serbia; ^4^Institute of Physiology, Medical Faculty Pristina, Anri Dinana bb, 38220 Kosovska Mitrovica, Serbia

## Abstract

In bacterial bone infections, excessively formed oxidants may result in local and systemic oxidative stress. Vitamin C is the major extracellular nonenzymatic antioxidant, also implicated in bone cells metabolism and viability. The physiological functions of vitamin C largely depend on its redox status. We sequentially assessed oxidative stress markers, hydroperoxides and malondialdehyde (MDA), total antioxidant activity (AOA), total vitamin C, ascorbic acid (Asc), and oxidized/reduced vitamin C ratio in 137 patients with acute osteomyelitis (OM). Compared to 52 healthy controls, in OM group baseline serum hydroperoxides, MDA and oxidized/reduced vitamin C ratio were higher whilst Asc and AOA were lower (*P* < 0.05, resp.). On the other side, total vitamin C levels in patients and controls were similar (*P* > 0.05), thereby suggesting a relative rather than absolute vitamin C deficiency in OM. During the follow-up, oxidative stress markers, AOA, and oxidizedreduced vitamin C ratio were gradually returned to normal, while there was no apparent change of total vitamin C concentrations. Persistently high values of oxidized/reduced vitamin C ratio and serum MDA were found in subacute OM. In conclusion, acute OM was associated with enhanced systemic oxidative stress and the shift of vitamin C redox status towards oxidized forms.

## 1. Introduction

Acute bacterial osteomyelitis (OM) is bone and bone marrow infection that is still associated with significant disability and poor clinical outcome [1]. In children, it is often caused by* Staphylococcus aureus* spread by hematogenous dissemination from some other site of inflammation and, occasionally, from inflamed neighboring tissues or by direct penetration through the open fracture wound. The disease is accompanied by synthesis of proinflammatory cytokines, activation and mobilization of phagocytic cells, thrombosis, necrosis, bone sequestration, and formation of a new bone at the site of infection.

It is well known that polymorphonuclear leukocytes (PMN) play important roles in limitation and resolution of bacterial bone infections. However, activated PMN synthesize highly reactive oxidants, like hydrogen peroxide, superoxide anion radical and hypochlorous acid, which may damage the bone tissue and cartilage [2, 3]. These oxidants were shown to increase the catabolic rate of bone matrix proteins, induce osteoclastogenesis and bone resorption, and suppress differentiation of osteoblastic and marrow stromal cells [4–9]. In biological systems, the concentration of oxidants is normally low and under strict control of enzymatic and nonenzymatic antioxidants. However, when defense mechanisms are overwhelmed, due to intrinsic deficiency, excessive formation of oxidants, or combination of both, oxidative stress occurs. Moreover, increased oxidative stress was previously reported in OM and some other osteoarticular diseases [2, 3, 10].

Vitamin C is the major nonenzymatic water-soluble antioxidant [11] and a regulator of bone cells metabolism and viability. In connective tissues, vitamin C serves as a cofactor of lysine and proline hydroxylase, implicated in collagen synthesis. In vitro studies suggest that vitamin C can suppress activity and reduce the survival rate of osteoclasts [7] while enhancing the survival [8] and differentiation of osteoblasts [9]. Besides, administration of antioxidant vitamins A, E, and C was shown to accelerate bone healing after long-bone fixative surgery by diminishing oxidative injury and promoting osteogenic activity [12].

Vitamin C exists in a reduced form, as ascorbic acid (Asc) and as oxidized vitamin consisting of partly oxidized dehydroascorbic acid (DHA) and fully oxidized 2,3-diketogulonic acid. Vitamin C participates in oxidation-reduction (redox) reactions. Electrons from Asc are lost stepwise with the formation of unstable radical intermediate semidehydroascorbic acid, which subsequently dismutate yielding Asc and DHA. The DHA must enter the cells to be reduced back to Asc; otherwise it is degraded to biologically inactive 2,3-diketogulonic acid and excreted into urine. Vitamin C behaves as an electron donor; thus, its antioxidant property actually depends on redox status. Given that vitamin C may have protective roles in bone infection, this study has investigated oxidative stress and vitamin C redox status during the treatment of acute pyogenic OM.

## 2. Materials and Methods

### 2.1. Patients

This study enrolled children treated at the Clinics for General and Orthopedic Surgery, Medical Faculty Pristina (Kosovska Mitrovica), for acute bacterial OM. There were 137 eligible patients with bacteriologically proven OM. The control group consisted of 52 age- and sex-matched healthy subjects who underwent clinical and laboratory checkup at least 6 months after complete recovery from uneventful fracture healing. Informed consent was provided from the parent or guardian. Participants who developed chronic OM, those with methicillin-resistant staphylococcal infection as well as those receiving vitamin C or other antioxidant supplementation therapy, were excluded. This study was conducted in accordance with the Declaration of Helsinki and approved by the institutional review board of the Medical Faculty, Pristina.

Clinical diagnosis of OM was based on the presence of one or more of the following criteria [13]: clinical signs and symptoms suggestive for bone and/or joint infection (local swelling, pain, tenderness, and restricted motion of affected limb), with duration of illness for less than two weeks; isolation of bacteria in blood or tissue culture; surgical finding of suppuration in the bone and/or joint; radiological finding suggestive for OM (periosteal reaction, bone destruction). On admission, all patients received empirical antibiotic therapy (first generation cephalosporins and vancomycin), analgesics, and antipyretics. If indicated, antibiotic therapy was changed after antibiogram was obtained. Surgical treatment consisted of incision, trepanation, curettage, and drainage of the inflamed bone tissue. Acute OM was defined as clinical improvement achieved to the end of the 6th week of treatment; cases with improvement achieved between 6th and 8th week were referred as subacute OM.

### 2.2. Biochemical Methods

The blood was taken into tubes without or with EDTA as an anticoagulant. White blood cell count (WBC) and differential red blood cell count (RBC), erythrocyte sedimentation rate (ESR), and hemoglobin concentration were routinely measured in anticoagulated blood. Serum C-reactive protein (CRP) was determined by immunoturbidimetric method on Hitachi 902 chemistry analyzer (Roche Diagnostics GmbH, Mannheim, Germany). Oxidative stress markers and antioxidants were assessed sequentially, in daily fresh samples taken on admission, on 2nd and 3rd week of hospital stay, and at the end of hospital treatment.

#### 2.2.1. Determination of Serum MDA Concentration

Concentration of serum malondialdehyde (MDA), as a relatively stable lipid peroxidation adduct, was assessed in daily fresh samples, using a modified thiobarbituric acid method as we previously described [14]. The absorbance readings of pinkish colored quinoneimine were taken at 515 nm, 532 nm, and 555 nm, on an UV/VIS spectrophotometer (Safas 2, Monaco). After Allen's correction was made, the concentration of MDA was calculated using a molar extinction coefficient of *ε* = 1.56 × 10^5^× L × M^−1^ × cm^−1^.

#### 2.2.2. Assessment of Serum Hydroperoxides

Concentration of total serum hydroperoxides, which are unstable lipid peroxidation products, was measured by the ferrous-oxidation xylenol orange method, after reduction of preexisting peroxides with triphenylphosphine [15]. The resulting ferric-xylenol orange complex was measured at 560 nm and calibrated against hydrogen peroxide standard curve at the concentration range of 0–50 *μ*mol/L.

#### 2.2.3. Measurement of Serum Total Vitamin C and Ascorbic Acid Concentrations

Concentration of total vitamin C (Asc plus oxidized vitamin C forms) was measured by 2,4-dintrophenylhydrazine (DNPH) method [16]. In this method, cooper (II) sulphate oxidizes Asc present in the sample to DHA. Newly formed and preexisting DHA thereafter react with DNPH under acidic conditions (pH 1-2) to give bis-2,4-dinitrophenyl hydrazone ascorbate, with absorbance maximum at 520 nm. Total vitamin C was measured after sample deproteinization (60 g/L metaphosphoric acid; 2 mM disodium EDTA), using freshly prepared 2,4-dintrophenylhydrazine-thiourea-copper (II) sulphate working reagent. Aqueous solutions of ascorbic acid (6–120 *μ*mol/L) were used to construct the calibration curve.

Preexisting oxidized vitamin C (DHA plus 2,3-diketogulonic acid) was determined after sample deproteinization using a DNPH reagent in which cooper (II) sulphate was omitted. The concentration of Asc was calculated as the difference between total and preexisting oxidized vitamin C. The preexisting oxidized vitamin C to Asc concentration ratio (oxidized/reduced vitamin C ratio) was calculated for each sample.

#### 2.2.4. Determination of Serum Total Antioxidant Activity

Total antioxidant activity (AOA) was measured using the benzoate-based colorimetric method [17]. This method evaluates the nonenzymatic antioxidant protection from hydroxyl radicals, brought by physiological levels of uric acid, albumin, and pyruvate. The results were expressed as mmol/L of uric acid.

### 2.3. Statistical Methods

Data distribution and homogeneity of variance were tested by the Kolmogorov-Smirnov test. Differences between groups were analyzed by one-way ANOVA. Post hoc comparisons were done using Student's independent or paired samples* t*-test, as appropriate. Frequency data were tested by chi-square test. Relationship between variables was assessed by calculating the Pearson correlation coefficient. Statistical significance was set at *P* < 0.05.

## 3. Results

A total of 137 patients with bacterial OM and 52 age- and sex-matched control subjects were enrolled in the study. Basic demographical, clinical, and laboratory findings of OM and control group are presented in Table 1. Osteomyelitis was most frequently localized on long bones: femur (32.8%), tibia (16.0%), and humerus (7.3%).* Staphylococcus aureus* was isolated in the most cases (91.2%). About 40.0% patients (*n* = 55) were with positive anamnesis of previous trauma, frequently after strenuous physical activity or blunt injury. During 6 weeks of treatment, 110 patients (80.3%) were fully recovered; the others (*n* = 27) were treated up for 8 weeks and considered to develop the subacute OM. The mean hospital stay in acute OM was 32.4 ± 4.1 days and in subacute OM 43.2 ± 4.9 days.

Routine laboratory tests performed at the time of admission showed increased mean values of ESR, CRP, and WBC and decreased RBC and hemoglobin levels in OM group (Table 1). In comparison to controls, mean baseline values of serum hydroperoxides, MDA, and oxidized/reduced vitamin C ratio were increased, whilst Asc and AOA were decreased in OM group; mean concentrations of total vitamin C were fairly similar in OM and control groups (Table 1). There was a direct correlation between serum hydroperoxides and WBC count (*r* = 0.207; *P* = 0.015) and serum Asc and total AOA (*r* = 0.213; *P* = 0.012); the correlation between MDA levels and WBC was not significant (*r* = 0.105; *P* = 0.221).

Of all OM patients, 112 received surgical treatment followed by antibiotics; the others (*n* = 25) were conservatively treated. There were no differences between surgically and conservatively treated groups regarding baseline oxidative stress markers, AOA, total vitamin C, and ascorbic acid as well as oxidized/reduced vitamin C ratio (*P* > 0.05, resp.).

**Figure 1:**
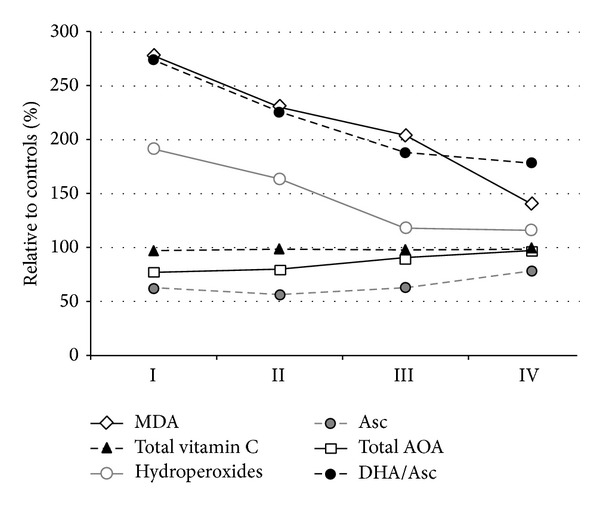
The time-course of serum hydroperoxides and MDA, total AOA, total vitamin C, Asc, and oxidized/reduced vitamin C ratio (DHA/Asc) in acute bacterial OM, assessed on admission (I), on 2nd week (II), on 3rd week (III) and at the end of hospital stay (IV). The results are expressed relatively to control values.

Table 2 and Figure 1 depict changes of oxidative stress markers and antioxidants during the treatment of OM. As shown, the concentration of serum hydroperoxides decreased while total AOA increased after two weeks of treatment. The concentration of serum MDA was slowly decreasing during the first two weeks and was still above control values at the end of follow-up. There was no apparent change in total vitamin C concentration. Instead, initially low concentration of Asc reached its minimum on the 2nd week and thereafter gradually increased, while oxidized/reduced vitamin C ratio was steadily decreasing. In conservatively treated group (*n* = 25), the reversal of oxidized/reduced vitamin C ratio was slower than that in surgically treated group (*n* = 112), and at discharge the difference was significant (1.43 ± 0.37 *μ*mol/L versus 1.67 ± 0.42 *μ*mol/L; in surgically versus conservatively treated groups; *P* = 0.005).

We further evaluated differences between acute and subacute OM groups (Table 3). At the baseline, serum hydroperoxides (*P* = 0.70), MDA (*P* = 0.48), AOA (*P* = 0.25), total vitamin C (*P* = 0.61), and Asc (*P* = 0.93) showed no differences between acute and subacute OM groups, while oxidized/reduced vitamin C ratio was higher in the latter group (*P* = 0.007). At the end of hospital stay, concentrations of serum MDA (*P* < 0.001), hydroperoxides (*P* = 0.013), and oxidized/reduced vitamin C ratio (*P* = 0.015) ratio were lower, whilst total AOA (*P* = 0.003) and Asc (*P* < 0.001) were higher in acute than those in subacute OM group (Table 3).

## 4. Discussion

The major finding of this study is that acute OM was associated with increased oxidative stress coupled with apparently normal serum total vitamin C levels, consisting mostly of oxidized vitamin forms. The disturbed vitamin C redox status was not improved during clinical treatment and worsened upon development of subacute OM. Consistently with previous reports [18, 19], the routine laboratory inflammation markers, such as WBC, ESR, and CRP, were increased already at the time of diagnosis, with approximately 76% of PMN in differential. There is considerable evidence that oxidant-mediated PMN bactericidal activity is necessary for limitation and resolution of bone infections. For example, subjects with chronic granulomatous disease, whose PMN lack functional NADPH oxidase and produce less reactive oxygen species than normal, may have high prevalence of OM [20]. Still, PMN-derived oxidants have low selectivity and can damage a variety of molecules leading to inflammatory bone destruction.

Like many other tissues, oxidants are constitutively formed at low levels in the bone to serve different regulatory functions and maintain the fine balance between osteoclast and osteoblast activity [21]. However, excess of oxidants can disrupt signaling pathways and promote differentiation and activation of osteoclasts, impair osteoblasts function, increase apoptosis, and depress osteogenesis, thereby resulting in bone resorption [5, 21]. Due to high levels of IL-6 or polymorphic* bax *gene expression, PMN may have prolonged lifespan in OM [22] and can therefore become a significant source of oxidants in circulation. Acting together with local factors, such as decreased blood flow, thrombosis, hypoxia, and expression of inducible cyclooxygenase [23], PMN may enhance the formation of oxidants in inflamed bone tissue.

Oxidants can induce lipid peroxidation of membrane-bound and plasma lipoprotein polyunsaturated fatty acids yielding various organic hydroperoxides. These compounds are chemically unstable and subsequently decompose to more stable MDA and other aldehydes, which can be toxic to osteoblasts [24]. Moreover, oxidative stress markers were increased at the time of diagnosis of OM in previous studies [10, 25–27], and our results confirm these reports. We also observed that hydroperoxides levels were normalized after 2nd week of treatment, whilst MDA was still above control values at discharge, particularly in subacute OM group, indicating that the intensity of oxidative stress could be related to the clinical course and complications of disease. Likewise, the higher serum MDA was recently associated with poorer prognosis in sepsis [28].

Systemic oxidative stress in OM was previously linked with decreased antioxidant enzymes paraoxonase and arylesterase [27], and total vitamin C [26]. In the current study, the resistance to oxidants, expressed as total AOA, was initially low in OM group and gradually returned to normal after three weeks of treatment. Estimation of AOA was based on ability of several antioxidants other than vitamin C, mainly albumin and uric acid, to prevent oxidative damage of serum biomolecules exposed to in vitro hydroxyl radical generating system [17]. In the current study, the concentration of serum total vitamin C in OM group was within the range of healthy controls and opposite from findings in adult OM patients [26]. Since we employed the same method to measure total vitamin C, this discrepancy probably reflected different age, dietary habits, or socioeconomic status of participants. However, we observed severely disturbed vitamin C redox status in OM group, with oxidized forms prevailing during the treatment, especially in subacute OM, pointing to imbalanced utilization/regeneration rates, rather than vitamin C deficiency.

During antioxidant reactions, Asc is rapidly oxidized to DHA and removed from the blood. This would sharply decrease its serum levels, as in critically ill patients following the onset of sepsis [29], and could underlie low baseline serum Asc in OM group in the current study. Activated PMN can play significant role in vitamin C recycling by reducing a wide range of DHA back to Asc over a short period of time [30]. However, this highly effective mechanism may lead to accumulation of Asc within the PMN resulting in delayed apoptosis [31], as reported in OM [22]. On the other side, excessive formation of DHA in vascular compartment during bacterial infection may cause the saturation of transporting systems or intracellular vitamin C reducing enzymes with the substrate [32], thereby contributing to the shift of serum vitamin C to more oxidized forms.

Oral vitamin C supplementation is commonly practiced all over the world, but its clinical relevance is still controversial even in pathologies associated with increased formation of oxidants [32, 33]. Experimental studies suggest that acting as a sacrificial antioxidant Asc can completely prevent oxidative modifications of various blood molecules [11]. However, Asc may also act as a prooxidant and sustain the production of oxidants, particularly during bacterial infections. In fact, Asc is required for conversion of compound III myeloperoxidase into biologically active compound I [34] and activated PMN accumulate millimolar levels of vitamin C [33] in order to maintain the synthesis of hypochlorous acid. Besides, parenteral administration of Asc during acute inflammation failed to protect extracellular molecules against myeloperoxidase- and other PMN-derived oxidants [35].

Vitamin C is thought to be highly protective to the bone by promoting osteoblast differentiation and activity and provide the stability of bone matrix [7–9]. Evidences suggest that Asc can ameliorate xenobiotic-induced toxicity at systemic levels [33] as well as the bone tissue [36]. Studies on fracture healing were less conclusive, and although Asc supplementation improved the mechanical resistance of fracture callus in elderly vitamin C genetically deficient rats, no histological or clinical improvements were seen in wild-type animals [37, 38].

The question that arises is whether vitamin C can influence the incidence and clinical outcome of bacterial bone infections. Blood and tissue concentrations of vitamin C in humans depend on diet. The higher vitamin C intake was reported to reduce the risk of bone marrow lesions and development of osteoarthritis in middle-aged subjects [39] and hip fracture in osteoporosis [40]. Under oxidative stress conditions, Asc may support osteoblastogenesis [41], implicated in bone regeneration and healing process. High levels of Asc combined with staphylococcal enterotoxin C may increase the differentiation rate of bone marrow-derived mesenchymal stem cells into osteoblasts [42]. These results, together with the linkage between subacute OM and lower serum Asc found in our study, point that Asc may have some favorable effects on bacterial bone infections. Still, Asc can act as a powerful prooxidant, and this feature may become important in bacterial infections.

As presented in the current study, patients with OM were under enhanced oxidative stress, corroborated by increased serum total hydroperoxides and MDA, and decreased total nonenzymatic antioxidant activity. The intensity of oxidative stress gradually returned to normal during the treatment, in accordance with the clinical course of disease. This was not accompanied by decreased total vitamin C levels but by significant and persistent shift of vitamin redox status towards oxidized forms. These results suggest an imbalance between oxidation and regeneration of Asc, rather than vitamin C deficiency. Given that Asc has multiple roles in the bone and that PMN may participate both in tissue damage and Asc recycling, further studies are needed to evaluate the metabolism of vitamin C during OM.

## Figures and Tables

**Table 1:** Basic demographical, clinical, and laboratory characteristics of patients with acute bacterial osteomyelitis and healthy controls.

	Patients (*n* = 137)	Controls (*n* = 52)
Age (years)	7.1 (6.6–7.6)	7.3 (6.3–8.3)
Gender (males/females; *f*)	85/52	32/20
Duration of illness (days)	4.6 (4.3–5.0)	NA
Recent trauma (yes/no; *f*)	48/89	NA
Causative agent (*f*):		
Staphylococcus aureus	125	NA
Streptococcus	4	
*Haemophilus influenzae *	2	
*Pseudomonas spp. *	2	
Other	4	
ESR (mm/hour)	68 ± 17	NA
CRP (mg/L)	51 (24–148)	NA
WBC (cells × 10^9^/L)	10.6 ± 2.5*	5.7 ± 1.4
PMN (cells × 10^9^/L)	7.5 ± 2.2*	3.2 ± 0.8
RBC (cells × 10^12^/L)	3.3 ± 0.4*	3.9 ± 0.9
Hemoglobin (g/L)	119 ± 27*	137 ± 22
MDA (*μ*mol/L)	2.83 ± 0.82*	1.02 ± 0.32
Hydroperoxides (*μ*mol/L)	12.04 ± 2.66*	6.31 ± 1.04
Total AOA (mmol/L)	1.54 ± 0.37*	2.01 ± 0.19
Total vitamin C (*μ*mol/L)	73.4 ± 15.1	75.5 ± 4.3
Ascorbic acid (*μ*mol/L)	26.2 ± 6.3*	42.0 ± 5.1
Oxidized/reduced Vit C	2.03 ± 0.48*	0.74 ± 0.20

Results are presented as mean value ± SD, geometric mean, and 95% confidence interval of the mean (in parenthesis) or frequency (*f*), obtained on admission to the hospital. NA: not applicable; *P < 0.05.

**Table 2:** Sequential changes and analysis of oxidative stress markers and vitamin C status during clinical treatment of acute bacterial osteomyelitis.

	Time of sampling			
	On admission	2nd week	3rd week	At discharge
MDA (*μ*mol/L)	2.83 ± 0.82	2.35 ± 0.66*	2.08 ± 0.60^∗¶^	1.44 ± 0.67^∗¶ §^
Hydroperoxides (*μ*mol/L)	12.04 ± 2.66	10.32 ± 2.16*	7.46 ± 2.23^∗¶^	7.32 ± 1.15^∗¶^
Total AOA (mmol/L)	1.54 ± 0.37	1.60 ± 0.40	1.81 ± 0.23^∗¶^	1.95 ± 0.32^∗¶ §^
Total vitamin C (*μ*mol/L)	73.4 ± 8.3	74.7 ± 10.2	74.2 ± 9.6	75.2 ± 9.7
Ascorbic acid (*μ*mol/L)	26.2 ± 6.3	23.5 ± 8.1*	26.4 ± 10.1^¶^	32.7 ± 6.3^∗¶^
Oxidized/reduced Vit C	2.03 ± 0.48	1.67 ± 0.63*	1.39 ± 0.54*	1.32 ± 0.35^∗¶^

Results are presented as mean value ± SD. Differences between groups were tested by one-way ANOVA and Student's *t*-test.

*P < 0.05 versus on admission.

^¶^ *P* < 0.05 versus 2nd week.

^§^ *P* < 0.05 versus 3rd week.

**Table 3:** Oxidative stress markers and vitamin C status in acute and subacute bacterial osteomyelitis.

	Acute osteomyelitis		Subacute osteomyelitis	
	On admission	At discharge	On admission	At discharge
MDA (*μ*mol/L)	2.80 ± 0.82	1.34 ± 0.42*	2.93 ± 0.83	1.84 ± 0.43^∗¶^
Hydroperoxides (*μ*mol/L)	11.9 ± 2.6	7.9 ± 1.1*	12.1 ± 2.8	8.5 ± 0.9^∗¶^
Total AOA (mmol/L)	1.56 ± 0.36	1.99 ± 0.31*	1.47 ± 0.39	1.79 ± 0.30^∗¶^
Total vitamin C (*μ*mol/L)	74.0 ± 8.2	75.7 ± 9.3	74.8 ± 10.1	75.0 ± 8.3
Ascorbic acid (*μ*mol/L)	26.2 ± 6.6	33.6 ± 3.9*	26.1 ± 5.1	29.0 ± 4.6^∗¶^
Oxidized/reduced Vit C	2.00 ± 0.54	1.27 ± 0.36*	2.32 ± 0.50^¶^	1.53 ± 0.37^∗¶^

Differences were tested by independent or paired samples Student's *t*-test.

*P < 0.05 on admission versus at discharge.

^¶^ *P* < 0.05 subacute versus acute OM.
